# MARK4 aggravates cardiac dysfunction in mice with STZ-induced diabetic cardiomyopathy by regulating ACSL4-mediated myocardial lipid metabolism

**DOI:** 10.1038/s41598-024-64006-7

**Published:** 2024-06-05

**Authors:** Yi Wu, Jingqi Zhang, Weiyi Wang, Dongdong Wu, Yang Kang, Lu Fu

**Affiliations:** 1 Laboratory of Cardiovascular Internal Medicine Department, The First Affiliated Hospital of Harbin Medical University, 23 Youzheng Street, Nangang District, Harbin, 150001 Heilongjiang China; 2 The First Affiliated Hospital of Jinzhou Medical University, 157 Renmin Street, Guta District, Jinzhou, 121000 China

**Keywords:** Cardiology, Diseases, Molecular medicine

## Abstract

Diabetic cardiomyopathy is a specific type of cardiomyopathy. In DCM, glucose uptake and utilization are impaired due to insulin deficiency or resistance, and the heart relies more heavily on fatty acid oxidation for energy, resulting in myocardial lipid toxicity-related injury. MARK4 is a member of the AMPK-related kinase family, and improves ischaemic heart failure through microtubule detyrosination. However, the role of MARK4 in cardiac regulation of metabolism is unclear. In this study, after successful establishment of a diabetic cardiomyopathy model induced by streptozotocin and a high-fat diet, MARK4 expression was found to be significantly increased in STZ-induced DCM mice. After AAV9-shMARK4 was administered through the tail vein, decreased expression of MARK4 alleviated diabetic myocardial damage, reduced oxidative stress and apoptosis, and facilitated cardiomyocyte mitochondrial fusion, and promoted myocardial lipid oxidation metabolism. In addition, through the RNA-seq analysis of differentially expressed genes, we found that MARK4 deficiency promoted lipid decomposition and oxidative metabolism by downregulating the expression of ACSL4, thus reducing myocardial lipid accumulation in the STZ-induced DCM model.

## Introduction

Diabetic cardiomyopathy is a disease that changes myocardial structure and function independently of diabetic macrovascular complications, and is mainly related to metabolic abnormalities in individuals with diabetes. Its pathogenesis is complex, and includes oxidative stress, myocardial energy metabolism disorders, inflammation and other mechanisms^1–6^. In recent years, an increasing number of studies have shown that increased myocardial lipid intake and accumulation are the main factors leading to the development of DCM^7,8^. However, there is currently no effective treatment tavailable for this disease, which has become the focus of related research in recent years.

The function of the microtubule affinity regulatory kinase (MARK) family was first characterized by the dissociation of microtubule- associated proteins from microtubules through phosphorylation, after which the members subsequently participated in microtubule dynamics. Earlier studies, revealed that the functions of the MARK family involved the regulation of the stability of the cell microtubule network and the regulation of cell polarity^9^. With further research, it was found that the MARK family can promote cell apoptosis, participate in glucose metabolism, and vascular bundle formation, and participate in energy homeostasis. Sun et al.^10^ reported that, through the upregulation of brown fat activity, MARK4 knockout mice exhibited excess appetite, increased activity and an increased metabolic rate to effectively prevent obesity caused by a high-fat diet. MARK4 deficiency can maintain glucose metabolism homeostasis in vivo by upregulating AMPK-related kinases. These data suggest that MARK4 may play a vital role in regulating glucose homeostasis and energy balance, suggesting that MARK4 may be a new drug target for treating metabolic diseases.

The long-chain acyl-CoA synthetase (ACSL) family comprises five subtypes, that catalyse ATP, coenzyme a (CoA), and long-chain fatty acids (LCFAs)^11^. Since LCFAs must be esterified to fatty acid (FA)-CoA to enter various metabolic pathways and then convert fatty acids into intracellular ester compounds through metabolic pathways and enter the oxidative catabolism of fatty acids to participate in fat metabolism, ACSLs are considered to be the rate-limiting enzymes in FA metabolism^12^. In the ACSL family, ACSL4 encodes an arachidonic acid-dependent isoenzyme 75 kDa in length that plays a crucial role in the metabolism of polyunsaturated fatty acids (PUFAs), especially arachidonic acid (AA)^13^. ACSL4 is distributed mainly in steroid synthesis tissues, such as the adrenal gland, ovary and testis, and its role in different metabolic pathways has been reported^14^. Several studies have shown that ACSL4 can participate in insulin secretion by pancreatic cells, and a decrease in ACSL4 expression is related to a decrease in insulin secretion caused by blood glucose stimulation. Knockdown of ACSL4 in adipocytes led to reduced incorporation of AA. Knocking down ACSL4 in the livers of adult mice fed a high-fat diet (HFD) ultimately resulted in a 43% reduction in hepatic arachidonoyl-CoA synthetase activity in the liver, while leading to a significant reduction in circulating VLDL-TG levels without affecting plasma cholesterol. In addition, the overexpression of ACSL4 led to increased fasting blood glucose and insulin levels in mice^15–17^. These results confirmed that ACSL4 plays an important role in lipid and glucose metabolism in hyperlipidaemic mice.

RNA sequencing (RNA-seq) can be used to analyse gene expression in prokaryotes and eukaryotes. With the continuous development of bioinformatics technology, we used RNA-seq to identify differentially expressed genes (DEGs), revealed the molecular functions and related biological pathways through analysis, and search for potential molecular targets and pathways^18^. In this study, we demonstrated for the first time that knocking down MARK4 in the STZ-induced DCM model improved lipid metabolism in mice, and high-throughput RNA-seq analysis was used to reveal the mechanism by which inhibition of MARK4 improves lipid metabolism by downregulating the expression of ACSL4.

## Results

### The expression of MARK4 was altered during STZ-induced diabetic cardiomyopathy-related injury both in vivo and in vitro

**Figure 1:**
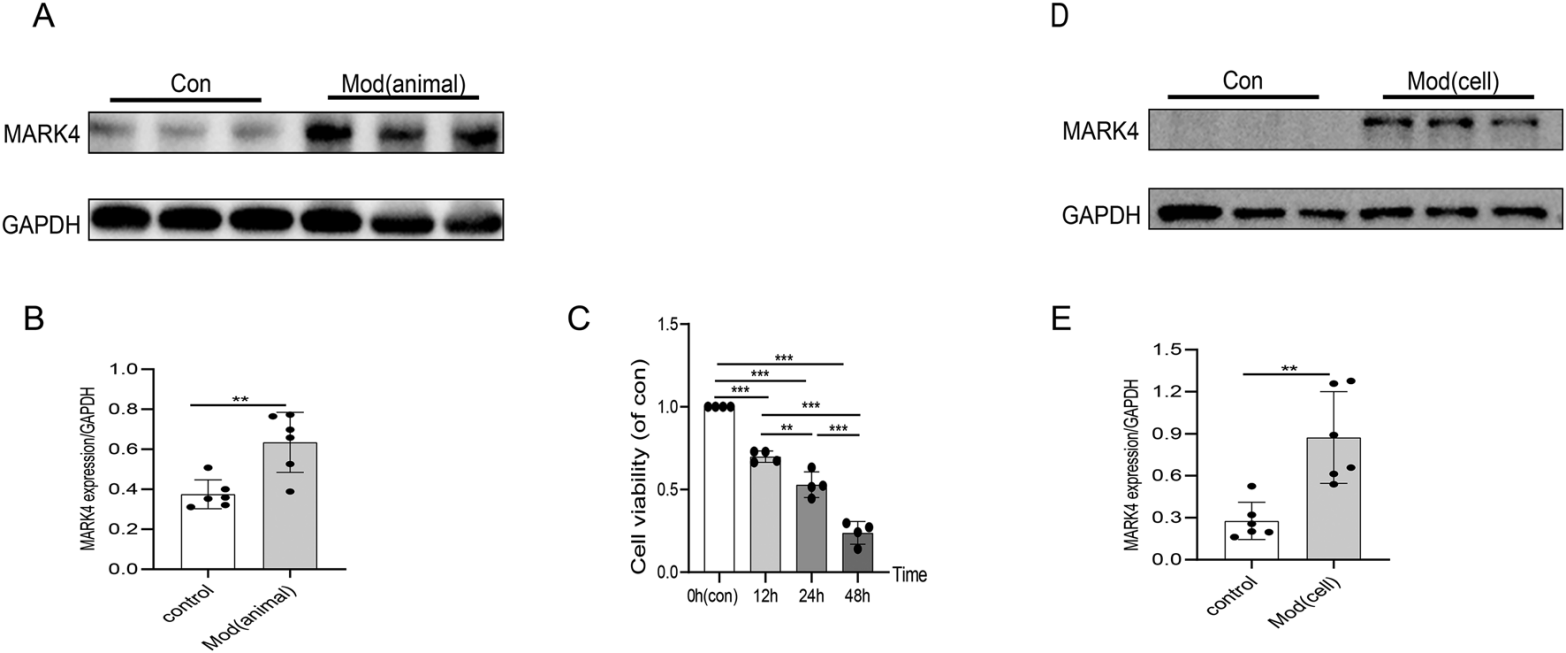
MARK4 was upregulated in vitro and in vivo. (**A**, **B**) The expression of the MARK4 protein in STZ-induced diabetic cardiomyopathy mice (n = 6 samples per group). (**C**) The effects of different durations of high-glucose and high-lipid treatment on cardiomyocyte viability (n = 4 samples per group). (**D**, **E**) The expression of the MARK4 protein in cardiomyocytes treated with high-glucose and high-lipid (n = 6 samples per group). The data are presented as the means ± SDs. Significant differences are indicated by *P < 0.05, **P < 0.01, and ***P < 0.001.

Western blotting was used to measure MARK4 expression in vivo and in vitro and to investigate the potential role of MARK4 in DCM. As shown in Fig. 1A,B, MARK4 expression was upregulated in STZ-induced DCM mice. Similarly, we found that MARK4 expression was also upregulated in cardiomyocytes stimulated with high glucose and high lipid levels in vitro (Fig. 1D,E). H9C2 cells were cultured in PA (200 μM) in vitro and in high-glucose culture medium (33.3 mM) for 0 h, 12 h, 24 h or 48 h to determine the optimal time point at which H9C2 cardiomyocytes were damaged by high glucose and lipid levels. The viability of H9C2 cells in each group was measured using the CCK-8 method, and the results showed that the viability of H9C2 cells decreased in a time-dependent manner in response to high glucose and high lipid levels. Compared with that at 0 h, the viability of H9C2 cells decreased significantly at 12 h, 24 h and 48 h (*P* < 0.01) (Fig. 1C). The results showed that high glucose levels combined with high lipid levels could reduce the viability of H9C2 cardiomyocytes, and 24 h was selected as the best time to stimulate H9C2 cardiomyocytes according to the cell viability value.

### Effect of MARK4 knockdown on body weight, blood glucose levels and oxidative stress in mice with STZ-induced DCM

**Figure 2:**
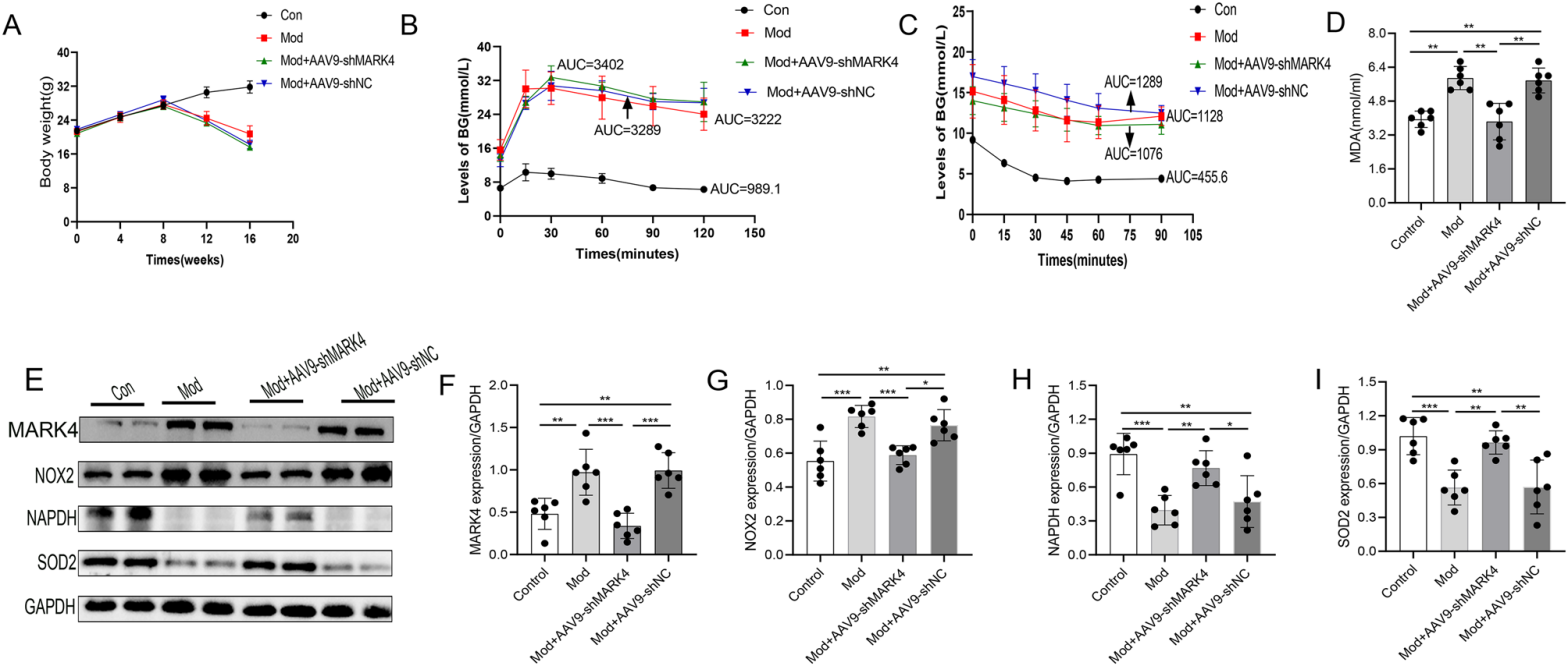
Effects of MARK4 deficiency on body weight, blood glucose levels and oxidative stress in STZ-induced DCM mice. (**A**) Body weight (n = 6 samples per group). (**B**) Intraperitoneal glucose tolerance test of blood glucose levels (n = 6 samples per group). (**C**) Intraperitoneal insulin tolerance test of blood glucose levels (n = 6 samples per group). (**D**) Levels of MDA in each group (n = 6 samples per group). (**E**–**I**) Western blot and quantitative analysis of the levels of oxidative stress-related proteins (NOX2, NAPDH, and SOD2) (n = 6 samples per group). The data are presented as the means ± SDs. Significant differences are indicated by **P < 0.01 and ***P < 0.001.

Weight change is a prominent feature of diabetes. As shown in Fig. 2A, the STZ injection started at 8 weeks, and the weight of the control group continued to increase, while the weights of the model group, model + AAV9-shMARK4 group and model + AAV9-shNC group all continued to decrease and were significantly lower than those of the control group. The characteristic of an increased blood glucose level in diabetic mice was observed via the intraperitoneal glucose tolerance test. The blood glucose level in the control group gradually increased from the beginning to the end of the experiment and then gradually decreased to normal, while the blood glucose levels in the other three groups rapidly increased from the beginning to the end of the study and then gradually decreased; however, the average blood glucose level was greater than 16 mmol/L, indicating impaired blood glucose regulation (Fig. 2B). The characteristics of insulin secretion in the mice were observed through an intraperitoneal insulin resistance test. The blood glucose level in the control group decreased rapidly and greatly, while that in the other three groups decreased slowly and less greatly, indicating that these three groups of mice exhibited insulin resistance (Fig. 2C). MDA is a product of lipid peroxidation, and its level can reflect the degree of damage to the body (Fig. 2D). Compared with that in the control group, a significantly greater serum MDA concentration was detected in the model group, indicating that oxidative stress occurred in the STZ-induced DCM mice. Compared with that in the model group, the MDA level was significantly lower after the AAV9-shMARK4 injection. The data showed that downregulating MARK4 in STZ-induced DCM mice could improve the oxidative stress response in vivo. Oxidative stress is an important mechanism for promoting the occurrence and development of DCM. We also investigated the effect of MARK4 on oxidative stress-related proteins (Fig. 2E). Compared with those in the control group, the NOX2 protein was upregulated in the model group, while the SOD2 and NAPDH proteins were downregulated. After the AAV9-shMARK4 intervention, the NOX2 level in STZ-induced DCM mice decreased, while the SOD2 and NAPDH levels increased (Fig. 2E–I).

### MARK4 knockdown improves cardiac function and myocardial tissue and mitochondrial structural damage in mice with STZ-induced DCM

**Figure 3:**
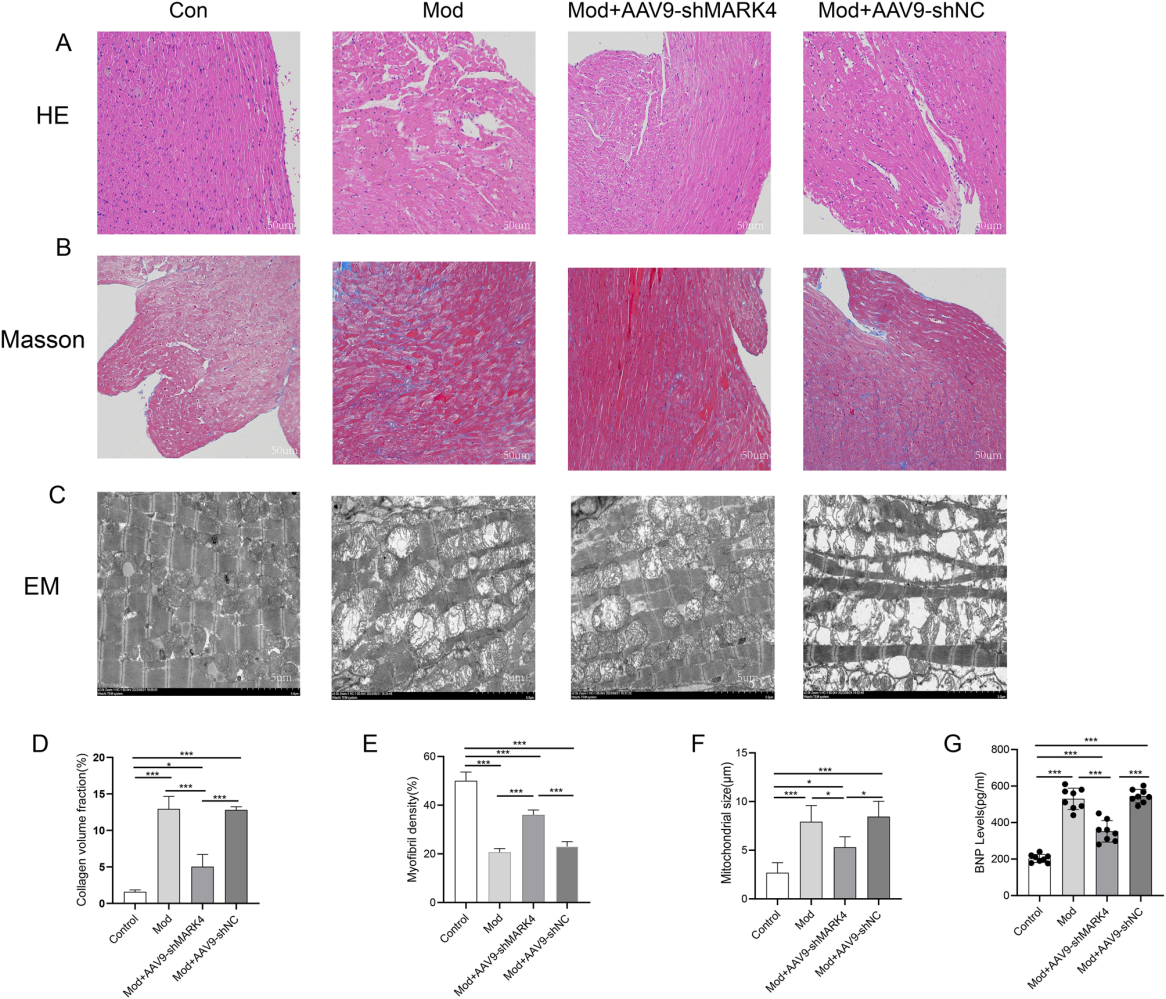
Effects of MARK4 deficiency on myocardial injury, myocardial fibrosis and mitochondrial structure in STZ-induced DCM mice. (**A**) Left ventricle stained with HE (original magnification × 200). (**B**) Left ventricle stained with Masson’s trichrome (original magnification × 200). (**C**) Transmission electron microscopy image of left ventricular myocardial (original magnification × 2000). (**D**) Quantitative analysis of collagen fibres using Masson’s trichrome staining (n = 3 samples per group). (**E**) Quantitative analysis of myofibrils in the myocardium using TEM (n = 3 samples per group). (**F**) Analysis of the mitochondrial size in the myocardium using TEM (n = 3 samples per group). (**G**) BNP levels in each group (n = 8 samples per group). The data are presented as the means ± SDs. Significant differences are indicated by *P < 0.05, **P < 0.01, and ***P < 0.001.

We examined the pathological structure and mitochondrial structure in myocardial tissue to investigate the effect of MARK4 deficiency on the myocardial structure in STZ-induced DCM mice. HE staining revealed that the myocardial fibres in the control group were tightly arranged and regular, and the nuclei were clearly visible. In the model group, cardiomyocytes exhibited extensive oedema, an increased cell space, unclear nuclei, and even fusion. After treatment with AAV9-shMARK4, the pathological changes in cardiomyocytes were significantly reduced, and the cardiomyocytes exhibited a more orderly arrangement (Fig. 3A). Myocardial fibrosis is considered an important feature of DCM. Masson’s trichrome staining was used to determine the degree of fibrosis. DCM-treated mice exhibited significant collagen accumulation (blue staining). Treatment with AAV9-shMARK4 significantly reduced the degree of fibrosis in the myocardial tissue (Fig. 3B,D). Mitochondrial morphology, and myofibril and mitochondrial size were observed using myocardial transmission electron microscopy (TEM), as shown in Fig. 3C,E and F. In the control group, myocardial fibres were arranged neatly, intercalated discs were clearly structured and complete, a large number of longitudinal and parallel myofibrils could be observed, mitochondrial cristae were arranged neatly and densely, with a normal structure, and the mitochondrial size was normal. Compared with those in the control group, the ultrastructure of the myocardium in the model group exhibited myocardial fibre running disorder, intercalated disc distortion and fuzzy fracture; the structure of each myotome zone was unclear; the mitochondria were obviously swollen; dissolution and vacuolation of local or whole mitochondrial ridge fractures occurred; and the nucleus and nuclear membrane were incomplete and presented segmental dissolution. After treatment with AAV9-shMARK4, the degrees of damage to the mitochondrial ultrastructure, mitochondrial size and myofibrillar destruction were significantly lower than those in the model group. The serum BNP level is an indicator of myocardial damage, and the higher its level, the more severe the myocardial damage (Fig. 3G). Compared with the level in the control group, the serum BNP levels in the model group and model + AAV9-shNC group were significantly increased, suggesting severe myocardial damage in both groups. After the AAV9-shMARK4 injection, BNP levels in mice were significantly reduced, indicating that inhibition of MARK4 expression could alleviate myocardial injury.

An echocardiogram revealed cardiac dysfunction due to diabetes. Compared with the values of the control group, the LVEF and LVFS of the model group were lower, and the LVIDs were higher, indicating impaired cardiac function in the STZ-induced DCM model mice. However, treatment with AAV9-shMARK4 improved the impaired cardiac function in STZ-induced DCM mice (Table 1).

**Table 1:** Effect of MARK4 knockdown on cardiac function in STZ-induced DCM mice (means ± SDs, n = 8).

Groups	LVEF(%)	LVFS(%)	LVIDd(cm)	LVIDs(cm)
Con	84.65 ± 3.79	47.61 ± 4.37	0.25 ± 0.03	0.13 ± 0.02
Mod	74.5 ± 2.72***	37.49 ± 2.35***	0.27 ± 0.02	0.17 ± 0.02**
Mod + AAV9-shMARK4	80.23 ± 4.08^##^	42.71 ± 3.91*^#^	0.24 ± 0.02	0.14 ± 0.02^##^
Mod + AAV9-shNC	75.68 ± 1.99***	38.44 ± 1.75***	0.26 ± 0.03	0.16 ± 0.02*

*P < 0.05, **P < 0.01, and ***P < 0.001 compared with the control group; ^#^*P* < 0.05 and ^##^*P* < 0.01 compared with the model group.

### MARK4 knockdown promoted mitochondrial fusion and inhibited myocardial apoptosis in mice with STZ-induced DCM

**Figure 4:**
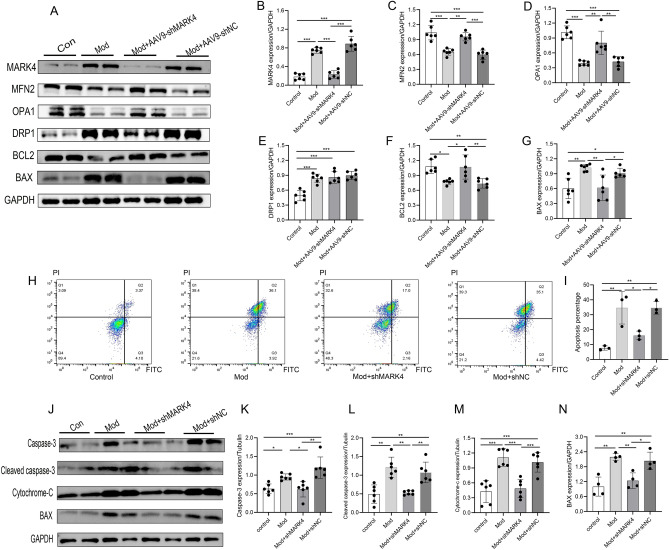
Effects of MARK4 deficiency on mitochondrial dynamics and myocardial cell apoptosis in STZ-induced DCM mice. (**A**–**G**) Western blot and quantitative analysis of the levels of mitochondrial dynamics- and apoptosis-related proteins (MFN2, OPA1, DRP1, BCL2, and BAX) (n = 6 samples per group). (**H**–**I**) Detection of the percentage of apoptotic cardiomyocytes in each group (n = 3 samples per group). (**J**–**N**) Western blot and quantitative analysis of apoptosis-related proteins (caspase-3, cleaved caspase-3, cytochrome c, and Bax) in cardiomyocytes (n = 6 samples per group). The data are presented as the means ± SDs. Significant differences are indicated by *P < 0.05, **P < 0.01, and ***P < 0.001.

Myocardial damage due to DM leads to increased mitochondrial fission, decreased fusion, and cardiomyocyte apoptosis. We also investigated the effects of MARK4 deficiency on mitochondrial dynamics and myocardial cell apoptosis (Fig. 4A–G). The expression of the mitochondrial fusion proteins MFN2 and OPA1 in the model group was significantly lower than that in the control group, and the expression of the fission protein DRP1 was significantly higher in the model group than in the control group. After treatment with AAV9-shMARK4, the expression of the mitochondrial fusion proteins MFN2 and OPA1 increased, but no significant difference was observed between the DRP1 and model groups. These data suggested that MARK4 deficiency does not affect mitochondrial fission induced by DCM but increases the protection of mitochondrial function by promoting mitochondrial fusion protein expression (Fig. 4A–E). As shown in Fig. 4F,G, the Bax protein was upregulated, and the Bcl-2 protein was downregulated in the model group. AAV9-shMARK4 decreased the level of Bax and increased the level of Bcl-2 in diabetic mice. The flow cytometry results were also consistent with the detection of apoptosis-related proteins (Fig. 4H–N). Compared with the control group, apoptosis was increased in the model group and model + shNC group, and the expression levels of the apoptosis-related proteins caspase-3, cleaved caspase-3, active bax and cytochrome-c were increased, while the expression levels of apoptosis and apoptosis-related proteins were decreased after the shMARK4 intervention. In conclusion, MARK4 deficiency can promote mitochondrial fusion and protect mitochondrial function while reducing myocardial cell apoptosis.

### MARK4 knockdown promotes fatty acid oxidation and decreases lipid levels in mice with STZ-induced DCM

**Figure 5:**
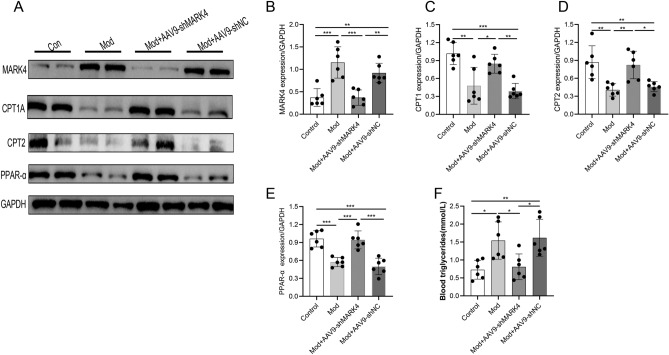
Effects of MARK4 deficiency on fatty acid oxidation and blood lipid levels in STZ-induced DCM mice. (**A**–**E**) Western blot and quantitative analysis of the levels of fatty acid oxidation-related proteins (CPT1A, CPT2, and PPAR-α) (n = 6 samples per group). (**F**) Blood triglyceride levels in each group (n = 6 samples per group). The data are presented as the means ± SDs. Significant differences are indicated by *P < 0.05, **P < 0.01, and ***P < 0.001.

Abnormal lipid metabolism in cardiomyocytes is an important cause of DCM. The aim of this study was to investigate the effects of MARK4 deficiency on myocardial lipid metabolism. We examined the expression of proteins associated with lipid oxidative metabolism (Fig. 5A). CPT1A, CPT2 and PPAR-α, key proteins involved in lipid metabolism, were expressed at significantly lower levels in the model group than in the control group. After the AAV9-shMARK4 treatment, the expression of these proteins increased. These findings suggested that the AAV9-shMARK4 treatment promoted lipid oxidative metabolism (Fig. 5A–E). The determination of the serum triglyceride concentration showed that the triglyceride level in the model group was significantly higher than that in the control group, but the triglyceride level in the model + AAV9-shMARK4 group was significantly lower than that in the model group (Fig. 5F). In summary, these data suggest that MARK4 deficiency can improve the abnormal lipid metabolism of cardiomyocytes induced by DCM, promote the oxidative breakdown of lipids, and reduce the myocardial accumulation of lipids.

### MARK4 knockdown affects lipid metabolism-related pathways in a diabetic cardiomyopathy model

**Figure 6:**
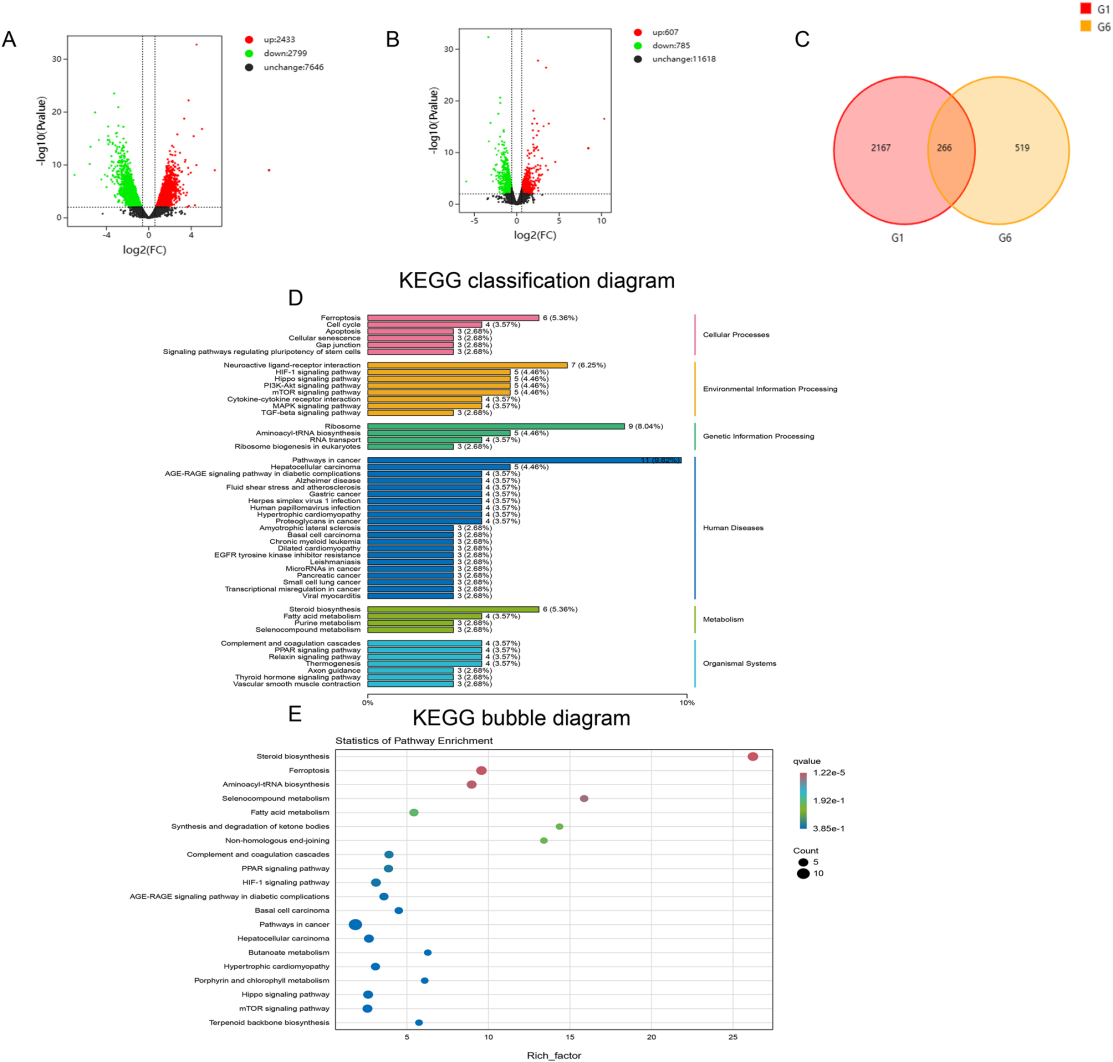

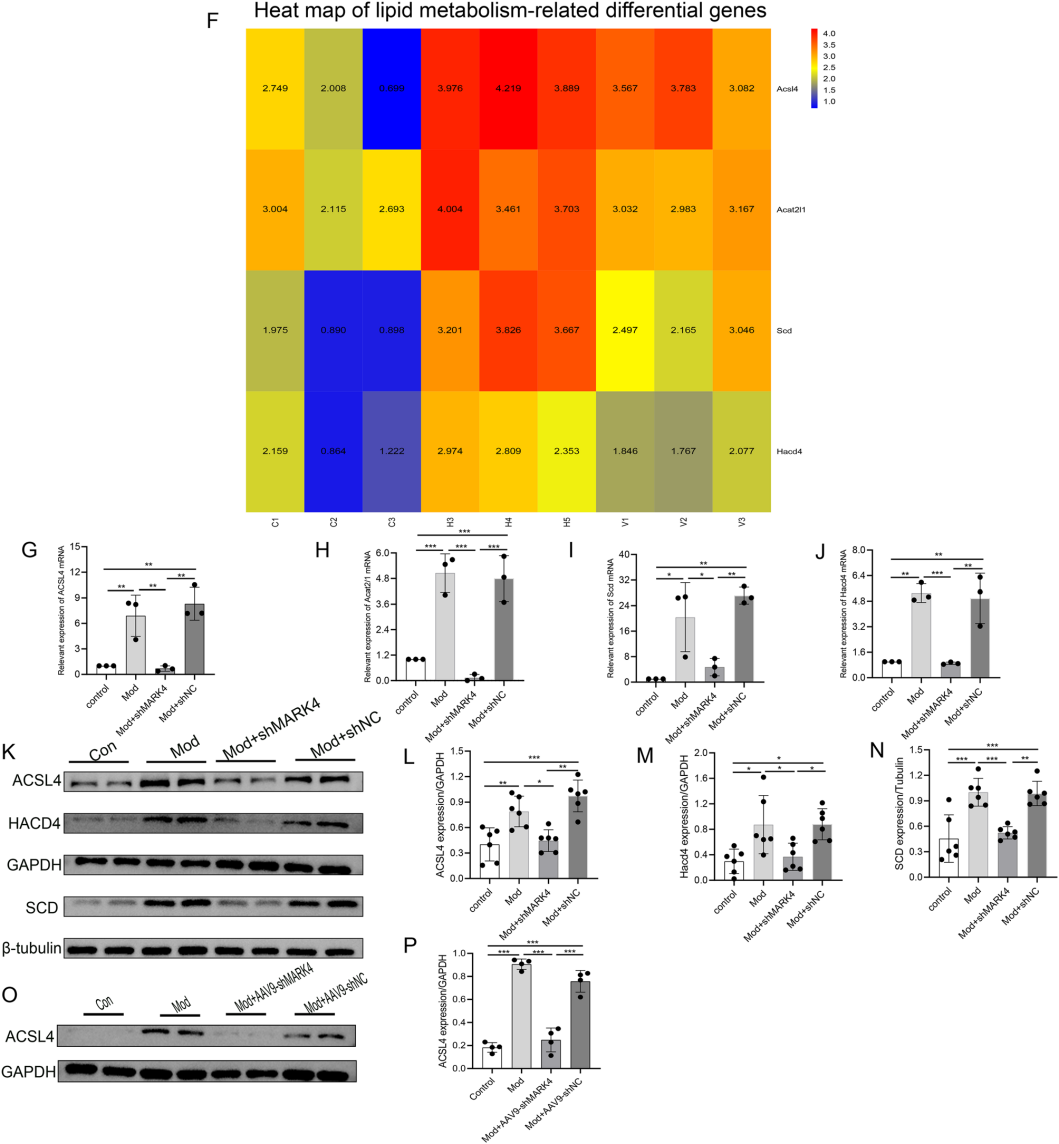
Transcriptomic analysis and sequencing validation of the effects of MARK4 deficiency. (**A**) Volcano plot of the control group and Hp + PA group (n = 3 samples per group). (**B**) Volcano plot of the Hp + PA group and Hp + PA + shMARK4 group (n = 3 samples per group). (**C**) The upregulated genes in the control group and Hp + PA group and downregulated genes in the Hp + PA group and Hp + PA + shMARK4 group are shown as a cross-Venn diagram (n = 3 samples per group). (**D**) KEGG classification diagram of the differentially expressed genes (n = 3 samples per group). (**E**) KEGG bubble diagram of the differentially expressed genes (n = 3 samples per group). (**F**) Heatmap of differentially expressed genes (n = 3 samples per group) (The heatmap analysis software: DESeq2(1.30.1) and edgeR(3.32.1) https://cran.r-project.org/web/packages/pheatmap). (**G**–**J**) Consistency of the qRT‒PCR and RNA-seq results (n = 3 samples per group). (**K**–**N**) Consistency of the Western blot and RNA-seq results (n = 6 samples per group). (**O**, **P**) Quantitative analysis of the ACSL4 protein levels in STZ-induced DCM mice with reduced MARK4 expression (n = 4 samples per group). The data are presented as the means ± SDs. Significant differences are indicated by *P < 0.05, **P < 0.01, and ***P < 0.001.

We performed RNA sequencing on H9C2 cardiomyocytes. The cells were divided into a control group, a Hg + PA group, and a Hg + PA + shMARK4 group for next-generation RNA sequencing. A total of 5232 DEGs was detected in the control group and Hg + PA group (2433 upregulated DEGs and 2799 downregulated DEGs) (Fig. 6A). A total of 1392 DEGs were detected in the Hg + PA group and Hg + PA + shMARK4 group (607 upregulated DEGs and 785 downregulated DEGs) (Fig. 6B). A total of 266 cross-genes were found by performing a cross-analysis of the upregulated genes in the control group and Hg + PA group and the downregulated genes in the Hg + PA group and Hg + PA + shMARK4 group (Fig. 6C). KEGG analysis^20–22^ and pathway annotation of 266 DEGs revealed that four DEGs were enriched in pathways related to lipid metabolism (Fig. 6D,E). These four genes were ACSL4, ACAT2/1, SCD, and HACD4. These genes were significantly downregulated in the Hg + PA + shMARK4 group (Fig. 6F). We verified the results for these 4 genes by performing real-time quantitative PCR and Western blotting to verify the accuracy of the RNA-seq results and the effect of the shMARK4 intervention on lipid metabolism in a DCM model. Compared with those in the control group, the expression of the lipid metabolism genes ACSL4, ACAT2/1, SCD, and HACD4 was significantly upregulated in the Hg + PA group. However, after shMARK4 treatment, the expression of these genes was downregulated relative to those in the Hg + PA group (Fig. 6G–J). The Western blot results were consistent with the above-described qRT‒PCR results (Fig. 6K–N). Subsequently, we detected the expression of ACSL4 in STZ-induced DCM mice with reduced MARK4 expression and found that the results were also consistent with the sequencing results (Fig. 6O–P).

### ACSL4 knockdown promotes lipid metabolism in the STZ-induced DCM model in vivo and in vitro

**Figure 7:**
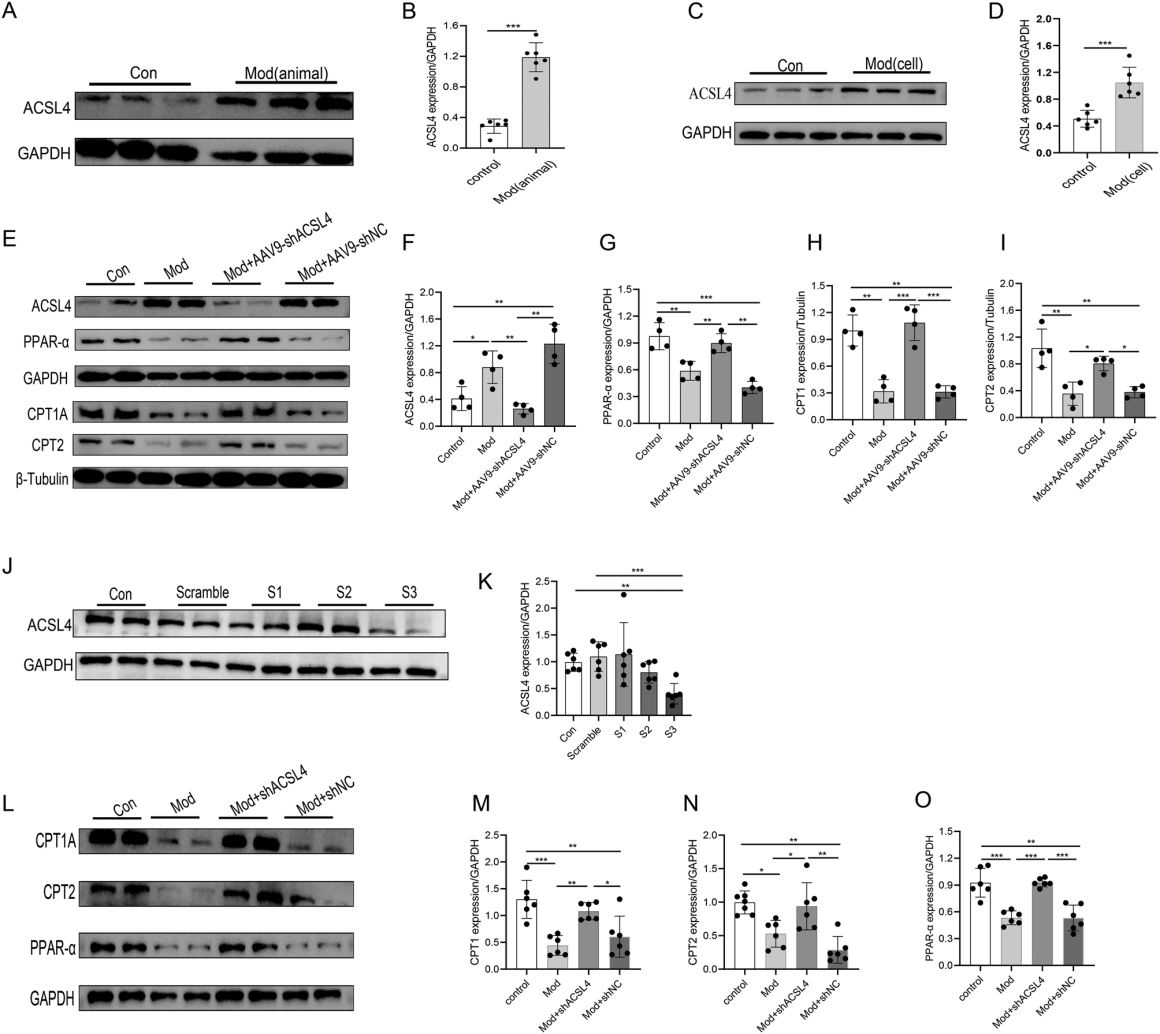
Expression of ACSL4 in vivo and in vitro in STZ-induced DCM models and the effect of ACSL4 deficiency on lipid metabolism in STZ-induced DCM models. (**A**, **B**) The expression of the ACSL4 protein in STZ-induced diabetic cardiomyopathy mice (n = 6 samples per group). (**C**, **D**) The expression of the ACSL4 protein in cardiomyocytes treated with high-glucose and high-lipid (n = 6 samples per group). (**E**–**I**) Western blot and quantitative analysis of the levels of fatty acid oxidation-related proteins (CPT1A, CPT2, and PPAR-α) following ACSL4 knockdown in STZ-induced DCM mice (n = 4 samples per group). (**J**, **K**) Changes in the expression of the ACSL4 protein in each group (n = 6 samples per group). (**L**–**O**) Western blot and quantitative analysis of the levels of fatty acid oxidation-related proteins following ACSL4 knockdown in DCM cardiomyocytes (n = 6 samples per group). The data are presented as the means ± SDs. Significant differences are indicated by *P < 0.05, **P < 0.01, and ***P < 0.001.

We examined the expression of the ACSL4 protein in the STZ-induced DCM model group and found that ACSL4 expression was significantly elevated in both the in vivo and in vitro DCM model groups compared with that in the control group (Fig. 7A–D). We then downregulated ACSL4 expression in STZ-induced DCM mice to examine its effect on the levels of proteins related to cardiac lipid oxidative metabolism (Fig. 7E). Compared with the control group, the expression of CPT1A, CPT2 and PPAR-α in STZ-induced DCM mice was significantly decreased, and the expression of these proteins increased after treatment with AAV9-shACSL4 (Fig. 7E–I). We constructed three shRNAs targeting ACSL4 and selected S3, which had the highest knockdown efficiency, for subsequent cardiomyocyte experiments to further verify the effect of the reduced expression of ACSL4 on cardiac lipid metabolism in cardiomyocytes in vitro (Fig. 7J,K). The results showed that compared with the levels in the control group, the expression of CPT1A, CPT2 and PPAR-α in the model group was significantly reduced, and the expression of these proteins was increased after the shACSL4 intervention (Fig. 7L–O). These data suggest that ACSL4 deficiency can promote the oxidative decomposition of lipids in the STZ-induced DCM model.

### Effects of ACSL4 overexpression after MARK4 downregulation on lipid metabolism, the mitochondrial membrane potential and reactive oxygen species levels in cardiomyocytes

**Figure 8:**
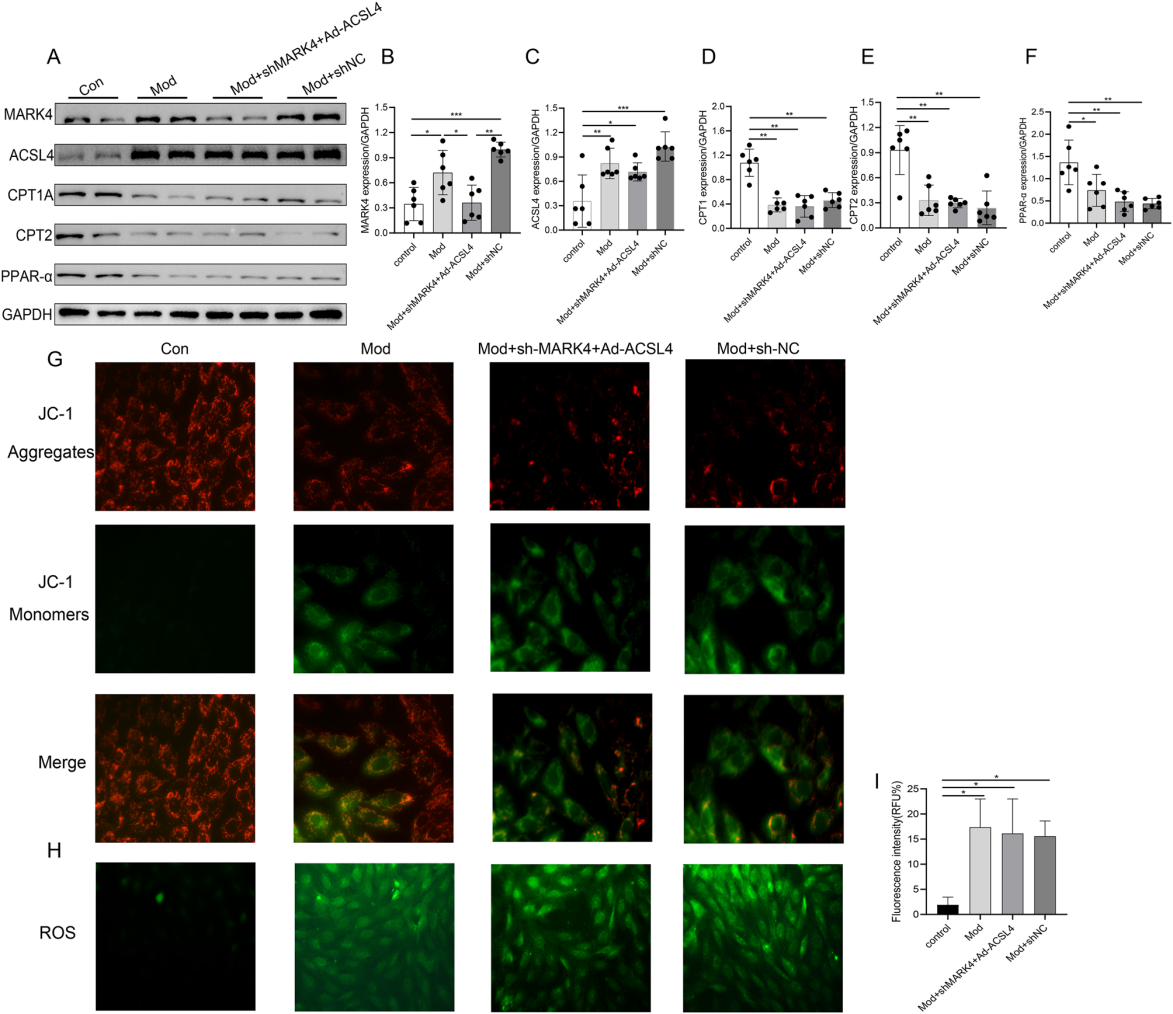
Relationships between MARK4 and ACSL4 and lipid metabolism. (**A**–**F**) Western blot and quantitative analysis of the levels of MARK4, ACSL4, CPT1A, CPT2, and PPAR-α (n = 6 samples per group). (**G**) Changes in the mitochondrial membrane potential in each group (n = 3 samples per group). (**H, I**) Changes in reactive oxygen species levels in each group (n = 3 samples per group). The data are presented as the means ± SDs. Significant differences are indicated by *P < 0.05, **P < 0.01, and ***P < 0.001.

Next, we increased ACSL4 expression in the context of MARK4 knockdown and detected the expression of these lipid oxidation proteins to confirm that MARK4 deficiency promotes lipid metabolism in the DCM model by downregulating ACSL4 expression (Fig. 8A). We found that, compared with the levels in the control group, the expression of CPT1A, CPT2 and PPAR-α in the model group was significantly lower, which was consistent with previous results. When ACSL4 expression was increased in the model group but was knocked down in the MARK4 group, the expression of CPT1A, CPT2 and PPAR-α was also significantly decreased, which was not significantly different from that in the model group (Fig. 8A–F). The mitochondrial membrane potential is an important indicator of mitochondrial function, and changes in reactive oxygen species levels can affect the physiological function of cells. We examined the effects of ACSL4 overexpression on the mitochondrial membrane potential and reactive oxygen species levels in cardiomyocytes in the absence of MARK4 by performing immunofluorescence staining. Compared with the control group, the mitochondrial membrane potential decreased significantly in the model group, model + sh-MARK4 + Ad-ACSL4 and model + sh-NC groups, and the production of reactive oxygen species increased significantly (Fig. 8G–I). These findings suggested that the expression level of ACSL4 is related to MARK4 expression, MARK4 protein deficiency can regulate lipid oxidation metabolism by downregulating ACSL4 expression, and overexpression of ACSL4 in the absence of MARK4 can increase the production of reactive oxygen species in cells and damage mitochondrial function.

## Discussion

**Figure 9:**
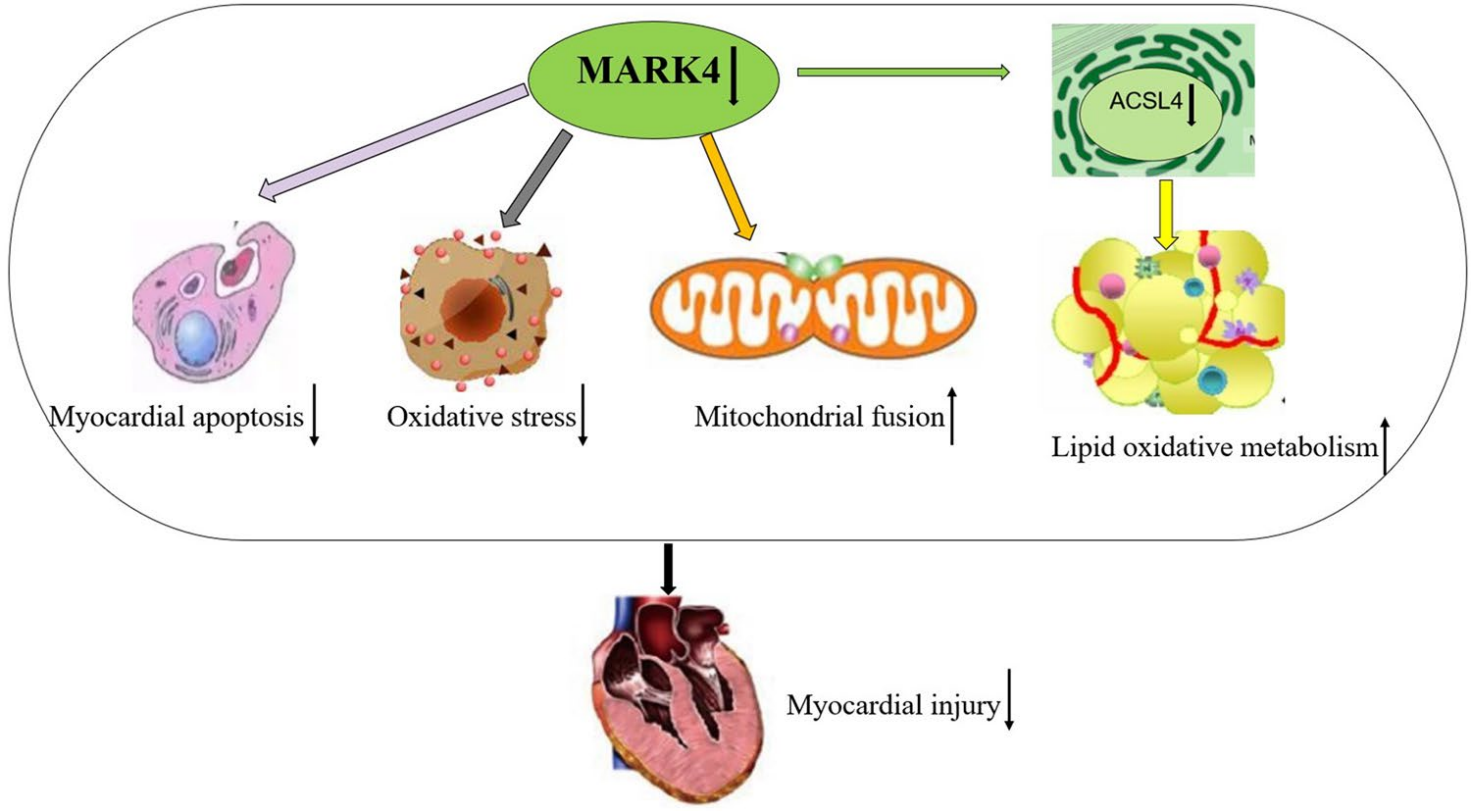
Schematic diagram of the mechanism of MARK4 deficiency improving myocardial injury in diabetes cardiomyopathy.

In this study, we found for the first time that MARK4 expression was increased in the STZ-induced model and that reducing MARK4 expression in the myocardia of DCM mice inhibited oxidative stress and myocardial apoptosis, promoted mitochondrial fusion and ACSL4-mediated lipid oxidative metabolism, and played a cardioprotective role (Fig. 9). These results indicate that MARK4 has the potential to become an effective target for the treatment of DCM.

MARK4 is located on chromosome 19 q13.2. This gene is 53,992 bp long and was originally known as microtubule-affinity regulated kinase analogue 1 (MAP/microtubule affinity-regulating kinase like 1, MARKL1). This gene is a member of the serine/threonine kinase family (MARKs) of cellular microtubule-associated proteins (MAPs) and plays an important role in regulating metabolism^23^. According to reports in the literature, mice in which MARK4 is specifically knocked out in of myocardial tissue exhibit significantly improved cardiac function after myocardial infarction, showing strong myocardial contractility close to that of healthy cardiomyocytes^24^. Other studies have shown that MARK4 knockout mice are less likely to suffer from obesity, which is mainly reduced by increasing the metabolic rate, to potentially reduce insulin resistance in mice and promote fat cell apoptosis, inflammation, and oxidative stress^25,26^. Although intraperitoneal glucose and insulin tolerance tests were also conducted in our study to detect the blood glucose levels of mice in each group, our results did not show that MARK4 knockdown could significantly improve the hyperglycaemic state and insulin resistance of DCM mice, which may be related to the short duration of our MARK4 intervention. In this study, both the model group and the model + AAV9-shMARK4 group exhibited hyperglycaemia. In this case, we still observed that the MARK4 intervention regulated myocardial lipid oxidation metabolism, but whether this regulatory effect is jointly affected by MARK4 and hyperglycaemia itself is unknown. We will extend the MARK4 intervention time or reduce the blood glucose level of DCM mice in subsequent studies to observe the effects of MARK4 on myocardial lipid oxidation metabolism under conditions of high and normal blood glucose levels. Other studies have shown that MARK4 can promote apoptosis, inflammation and oxidative stress in adipocytes, and these results reveal the physiological function of MARK4 in fat metabolism. In our study, the effects of MARK4 on myocardial apoptosis and oxidative stress in STZ-induced DCM model were also observed, and the results showed that inhibition of MARK4 expression could reduce myocardial oxidative stress and apoptosis. These results also revealed that MARK4 plays an important biological role in cardiomyocytes. However, we need to further explore the mechanism by which it regulates apoptosis and oxidative stress.

Fatty acids and glucose are the main substrates for myocardial energy metabolism, and under physiological conditions, fatty acid β-oxidation is the main energy source for the heart. Conversely, glycolysis predominates in response to pathological stimuli such as ischaemia and heart failure^27,28^. The oxygen consumption of the heart required for the production of adenosine triphosphate (ATP) is greater when fatty acids are used than when glucose is used. However, in diabetic hearts, fatty acid beta-oxidation increases and glucose oxidation decreases. Increased utilization of fatty acids in the diabetic heart is associated with reduced cardiac efficiency, which is a sign of diabetic cardiomyopathy^29–31^. Our study revealed that knockdown of MARK4 expression inhibited ACSL4 expression. Subsequently, through cell rescue experiments, we increased ACSL4 expression after the knockdown of MARK4 expression and observed a significant decrease in the expression of key proteins involved in lipid oxidative metabolism (CPT1A, CPT2 and PPAR-α), which was not significantly different from that in the model group. These findings suggest that the deletion of MARK4 is necessary to promote lipid oxidative metabolism in the STZ-induced DCM model by downregulating ACSL4 expression. In summary, our results confirmed that MARK4 knockdown can alleviate myocardial injury in the STZ-induced DCM model, promote lipid oxidative reactions and reduce lipid accumulation by downregulating ACSL4 expression.

The effect of MARK4 on glycolipid metabolism has been confirmed in previous studies, but its mechanism of action has not been fully elucidated. In this study, the RNA-seq analysis revealed that lipid metabolism was altered in the absence of MARK4. Further analysis of the differentially expressed the lipid metabolism-related genes ACSL4, SCD, ACAT2/1 and HACD4 revealed that their expression was inhibited in MARK4-knockdown cardiomyocytes. These downstream genes, whose expression is also reduced with the decrease of MARK4 expression, have important biological functions. HACD4 is a protein-coding gene involved in the biosynthesis and metabolism of fatty acyl-CoA, which is mainly associated with spinocerebellar ataxia and atherosclerosis in men^32^. ACAT2/1 is a membrane protein that is expressed in all tissues and cells throughout the body, and its main function is related to cholesterol metabolism^33,34^. SCD, an ER protein, is a rate-limiting enzyme that catalyses the production of monounsaturated fatty acids. It has four subtypes in mice, namely, SCD1, SCD2, SCD3 and SCD4. Each subtype is expressed mainly in different places, and SCD4 is expressed mainly in the heart. Studies have shown that insulin sensitivity and lipid metabolism are significantly improved after SCD1 gene knockout in mice fed a high-fat diet. In an experimental model of heart failure, the upregulation of SCD1 expression resulted in a decrease in the LVEF and the myocardial accumulation of lipids, which promoted the occurrence of cardiac dysfunction^35^. ACSL4 is a member of the long-chain acyl-CoA synthetase (ACSL) family, and ACSLs are considered rate-limiting enzymes in fatty acid metabolism^12^. ACSL4 is expressed mainly in peroxisomes, mitochondria and the endoplasmic reticulum, and it mediates catalytic synthesis of acyl CoA in vivo^14,36^. The inhibition of ACSL4 can reduce the damage caused by Parkinson's disease by reducing lipid and reactive oxygen species levels^37^. However, the exact role of ACSL4 in lipid oxidative metabolism in DCM has not been determined. We subsequently detected the expression of ACSL4 in the STZ-induced DCM model and found that ACSL4 expression was increased in the STZ-induced DCM model with consistent results in vivo and in vitro. By knocking down ACSL4 expression in the STZ-induced DCM model, we observed the effect of ACSL4 the levels of on vital proteins involved in lipid oxidative metabolism. Our results showed that the expression of key proteins involved in lipid oxidative metabolism (CPT1A, CPT2 and PPAR-α) was reduced in cardiomyocytes cultured with high glucose combined with palmitic acid, and the shACSL4 intervention reversed these changes, with consistent results in vivo and in vitro. This finding suggested that the downregulation of ACSL4 can promote the expression of lipid oxidative metabolism products and promote lipid oxidative decomposition.

In conclusion, although we found that MARK4 can affect myocardial lipid metabolism by regulating ACSL4 expression, based on the current data, the specific mechanisms underlying this regulation have not been fully clarified, and further studies are needed. In this study, we preliminarily found that the mouse STZ-induced DCM models exhibited increased myocardial oxidative stress and apoptosis and decreased myocardial lipid oxidative metabolism. However, after the AAV9-shMARK4 intervention, oxidative stress and apoptosis decreased in the mouse myocardium, and the oxidative metabolism of lipids was promoted by downregulating the expression of ACSL4. These results suggest that interventions targeting MARK4 expression may be an effective strategy for treating diabetic heart disease. However, there are some limitations in this study. The DCM model induced by low-dose STZ injection combined with high-fat diet has certain differences from other diabetic models. Although myocardial injury, myocardial apoptosis, increased oxidative stress, mitochondrial dysfunction, and impaired lipid metabolism in the DCM model have no significant differences from other diabetic models^38–47^. However, whether MARK4 intervention can also reduce myocardial apoptosis and myocardial oxidative stress, alleviate mitochondrial dysfunction and promote myocardial lipid metabolism in other diabetes models, which needs further research in the future.

## Materials and method

### Animal model

Adult male C57BL/6 J mice (4 weeks old) were purchased from the Experimental Animal Center of the Second Affiliated Hospital of Harbin Medical University. The mice were housed in a temperature-controlled room where they had access to plenty of water and food, unless otherwise indicated. The experiment was approved by the Research Ethics Committee of the First Affiliated Hospital of Harbin Medical University and was performed in accordance with the Experimental Animal Care and Ethics Committees of the First Affiliated Hospital of Harbin Medical University (IACUC:2021118), and confirming that all experiments were performed in accordance with ARRIVE guidelines. After 2 weeks of acclimatization–the mice were divided into a normal chow diet (D12450, Research Diets, United States) and a high-fat diet (D12492, Research Diets, United States) for 8 weeks. The HFD-fed mice were then intraperitoneally injected with freshly prepared streptozotocin (50 mg/kg, STZ; Sigma‒Aldrich, St. Louis, MO, USA) dissolved in 0.1 mM sodium citrate buffer (pH 4.5) for 5 consecutive days. The ND mice were injected with the same volume of sodium citrate buffer. Fasting blood glucose levels were evaluated 7 days after the final injection. A fasting blood glucose level greater than 16.7 mmol/L in three independent tests was considered to indicate successful establishment of the diabetes model, and these mice were recruited for subsequent experiments^8^. Both diabetic and control mice were continually fed either the HFD or ND for 4 weeks. The AAV9 vector was constructed with small hairpin RNA (shRNA) under the control of the cTnT promoter against MARK4 or ACSL4 (AAV9-shMARK4 or AAV9-shACSL4) or the corresponding negative control AAV9-shNC. Then, 12 weeks after the induction of diabetes, the diabetic mice were intravenously injected with AAV9-shNC or AAV9-shMARK4 or AAV9-shACSL4 ([5 × 10^11^ vp (viral particles)] per mouse), after which they were fed a HFD or ND for 4 weeks. The weights of the mice were measured every four weeks. After 16 weeks, the mice were euthanized using carbon dioxide asphyxiation and heart samples were taken. Accordingly, the mice were divided into the following groups: the control mice + vehicle (Con) group, the diabetic cardiomyopathy mice (Model) group, the diabetic cardiomyopathy mice + AAV9-shMARK4 (Model + AAV9-shMARK4) group, the diabetic cardiomyopathy mice + AAV9-shACSL4 (Model + AAV9-shACSL4) group and the diabetic cardiomyopathy mice + AAV9-shNC (Model + AAV9-shNC) group.

### Cell culture and RNA interference

H9C2 cells were purchased from the Chinese Academy of Sciences (Shanghai) and cultured in a 37 °C incubator with DMEM plus 10% foetal bovine serum and 5% CO_2_. To simulate hyperglycaemic and hyperlipid toxicity during the development of diabetic cardiomyopathy, we cultured H9c2 cells in 33.3 mM glucose (HG) and 200 μM palmitic acid (PA)^19^ for 12 h, 24 h, and 48 h. The CCK8 method was used to calculate cell viability, and the appropriate culture time was selected. MARK4 and ACSL4 were knocked down in the cells under the above culture conditions, and lentiviruses containing sh-MARK4 and sh-ACSL4 were used for 24 h at an infection multiple (MOI) of 50. After 24 h, the cell medium was replaced with the selected medium (whole medium + 5 μg/ml purinomycin or whole medium + 20 μg/ml neomycin). The transfected cells were preserved until the untransfected cells died.

### Cardiac echocardiography

Four weeks after virus injection induction, the cardiac function indices were measured using a Philips Sonos 5500 multifunctional colour ultrasound device (Philips, USA) and an 8 MHz transducer. The left ventricular internal diameter at end-diastole (LVIDd), left ventricular internal diameter at end-systole (LVIDs), left ventricular ejection fraction (EF), and fractional shortening (FS) were measured and recorded.

### Intraperitoneal glucose tolerance test (IPGTT)

The mice were fasted overnight and subjected to blood glucose tests after intraperitoneal injection of 2 g/kg glucose (Sigma‒Aldrich). Blood glucose levels were measured using an Accu-Chek glucose metre (Roche) at 0, 15, 30, 60, 90 and 120 min.

### Intraperitoneal insulin tolerance test (IPITT)

The mice were fasted overnight and subjected blood glucose test after intraperitoneal injection of 0.5 U/kg insulin. Blood sugar levels were measured at 0, 15, 30, 45, 60 and 90 min using the Accu-Chek glucose meter (Roche).

### Cell viability assay

Cell viability was measured using a CCK-8 (Beyotime, China) assay. The cultured cells were inoculated into 96-well plates at a density of 5 × 10^3^, and cultured with 33.3 mM glucose (HG) and 200 μM palmitic acid (HP) for 12 h, 24 h, or 48 h, respectively. Then, 10 µL of CCK-8 solution was added to each well, and the cells were incubated for 1.5 h at 37 °C. The absorbance at 450 nm was quantified with a Vector 5 (Bio-Tech Instruments, Winooski, VT, USA).

### H&E staining and Masson staining

After sampling, the heart was fixed in 4% paraformaldehyde solution, after which the heart samples were embedded in paraffin, after which continuous sections were obtained. The sections were then stained HE and Masson’s trichrome and observed under a microscope (Olympus BX51, Japan).

### Myocardial transmission electron microscope

After sampling, the heart was removed and put into 4% glutaraldehyde solution for embedding and solidification. The myocardial tissue was sliced into 5 µm sections with an ultrathin microtome and stained for 10 min. Finally, the cells were observed under a transmission electron microscope (Hitachi, Japan).

### Detection of apoptosis

An Annexin V-FITC/PI Apoptosis Kit (MULTI SCIENCES, China) was used. The fluorescence intensity was determined via flow cytometry BD Accuri C6 Plus, China) and analysed via FlowJo software.

### Determination of serum MDA, BNP and triglyceride

The malondialdehyde (MDA) concentration in the serum was determined by commercial assay kits (Nanjing Jiancheng BioTech. Co. Ltd., Jiangsu, China). The serum BNP levels were detected by ELISA kit (Jianglai Biology, Shanghai, China). The serum triglyceride concentration was detected via an automatic biochemical instrument (Wuhan Servicebio Technology. CO. Ltd., Wuhan, China).

### Real-time PCR

Total RNA was extracted from myocardial tissue or H9C2 cells with an RNA Extraction Kit (Takara Bio, Otsu, Japan), and cDNA was synthesized using a PrimeScript™ RT Reagent Kit with gDNA Eraser (Takara Bio, Otsu, Japan) according to the manufacturer’s instructions. The expression levels of candidate genes were measured with SYBR Green on an ABI 7500 Real-Time PCR System (Applied Biosystems, Foster, CA, USA). The following primer sequences were used for this experiment: MARK4: forward: 5′-TGAAGGGACTCAACCACCC-3′ and reverse: 5′-TCACCAGGTATAGCGTCTTCTC-3′; GAPDH: forward: 5′-GGCACA GTCAAGGCTGAGAATG-3′ and reverse: 5′-ATGGTGGTGAAGACG CCAGTA-3′. GAPDH was used as an internal standard. The 2 − ΔΔCT method was used to calculate the mRNA levels of each gene.

### Western blot

RIPA buffer (Beyotime, China) was used to harvest total protein. The protein concentrations were determined using a BCA Protein Assay Kit (Beyotime, China), and all the experiments were repeated at least three times.

Antibodies:

- MARK4:(#4834,1:1000, CST), NOX2(1290681:5000, Abcam),
- NAPDH:(133303,1:1000, Abcam), SOD2:(WL02506,1:500, Wanleibio, China), MFN2:(124773, 1:5000, Abcam), OPA1:(42364,1ug/ml, Abcam),
- DRP1:(156951, 1:2000, Abcam), BAX:(182733,1:2000, Abcam), BAX (6A7, MA5-14003,1:100, ThermoFisher)
- BCL-2:(196495,1:1000,Abcam), Cytochrome-C (T55734,1:1000,Abmart), Cleaved caspase-3 (19677-1,1:1000,Proteintech),Caspase-3 (19677,1:1000,Proteintech),CPT1A:(234111,1:1000,Abcam),CPT2:(181114,1:5000, Abcam), PPAR-α:(314112, 1:1000, Abcam), ACSL4:(22401-1-AP,1:5000, Proteintech, China), HACD4(DF15858,1:1000, Affinity), SCD:(28678–1-AP, 1:10000, Proteintech, China), GAPDH:(TA-08,1:5000, Zsbio), β-tubulin(AB0039,1:5000,Abways).

### Measurement of mitochondrial membrane potential

The treated cells were collected and the changes of mitochondrial membrane potential were observed by JC-1 staining. The cells were washed with PBS once, 1 ml of 10% FBS was added to the cell orifice plate, and 1 ml of JC-1 dyeing solution was added to it, and the two were mixed evenly and incubated in a cell incubator at 37 °C for 30 min to ensure successful staining of all cells. Finally, the cells were washed with 1 × JC-1 staining buffer, and an appropriate amount of 10% FBS was added to the cell pore plate. Finally, the cells were observed and photographed under a fluorescence microscope. The excitation wavelength of green fluorescence is 514–529 nm, and the excitation wavelength of red fluorescence is 585–590 nm.

### Measurement of active oxygen species in mitochondria

Intracellular ROS levels were measured using reactive oxygen species assay kit (Beyotime Biotechnology, China). According to the instructions, the treated cells were washed once with PBS, appropriate DCFH-DA solution was added, incubated at 37 °C for 20 min, and then observed under a fluorescence microscope (olympus), excited at 488 nm and emitted at 525 nm for fluorescence quantitative analysis.

### Transcriptome analysis

For RNA sequencing (RNA-seq) studies, H9C2 cells treated differently were quickly frozen at − 80 °C for further analysis. Total RNA was extracted from cells with TRIzol reagent. The sample quality was checked using a Nanodrop system and 6000 Assay Kit (Agilent Technologies, CA, USA). After the total RNA sample was quantified, the mRNA was reverse transcribed into double-stranded complementary cDNA. The purified double-stranded cDNA was subjected to end repair and PCR amplification to complete library preparation. The Illumina HiSeq X Ten platform (ANOROAD, Beijing, China) was used for sequencing to obtain the original reads. To make the estimated gene expression levels of different genes and experiments comparable, the expression values were normalized to fragments per thousand bases per million mapping reads (FPKM). GO and KEGG enrichment analyses of the differentially expressed genes were performed using R language. The screening of differentially expressed genes involved determining the fold change (FC) and q value as related indicators. Usually, DEGs with a | log2(fold change)|≥ 1 and q < 0.05 were considered to be significantly differentially expressed genes.

### Statistical analysis

All the data are expressed as the mean ± standard deviation (SD). Student t-test is used to calculate the difference between the two groups, and one-way ANOVA analysis is used for comparison between multiple groups. RNA-seq analysis was performed using BMKCloud (www.biocloud.net). Fluorescence quantification and pathological analysis quantification were performed using ImageJ Fiji. The data were analysed and graphed using GraphPad Prism software 8.0. *P* values < 0.05 were considered to indicate statistical significance.

### Ethics approval and consent to participate

This study was approved by the Research Ethics Committee of the First Affiliated Hospital of Harbin Medical University (IACUC:2021118). We confirming that all experiments were performed in accordance with ARRIVE guidelines.

## Data Availability

The datasets used and/or analyzed in the current study are available from the corresponding author on reasonable request.

## Abbreviations

DCM: Diabetic cardiomyopathy
MARK4: Microtubule affinity‐regulating kinase 4
AMPK: AMP-activated protein kinases
DM: Diabetes millitus
MARKs: Microtubule affinity regulatory kinases
LCFAs: Long-chain fatty acids
ACSL: Long-chain acyl-CoA synthetase
CoA: Coenzyme a
FA: Fatty acid
PLs: Phospholipids
TGs: Triglycerides
PUFA: Polyunsaturated FA
AA: Arachidonic acid
RNA-seq: RNA sequencing
DEGs: Differentially expressed genes
